# Using user-centered design to better understand challenges faced during genetic analyses by novice genomic researchers

**DOI:** 10.3389/fbinf.2026.1849524

**Published:** 2026-05-15

**Authors:** Harsh Vijaykumar Patel, Gail P. Jarvik, Taryn O. Hall, David L. Veenstra, Serena Jinchen Xie, David R. Crosslin

**Affiliations:** 1 University of Washington, Seattle, WA, United States; 2 Optum Whole Health Solutions, Minneapolis, MN, United States; 3 Biomedical Informatics and Genomics, Tulane University, New Orleans, LA, United States

**Keywords:** computational biology, genomics, novice researchers, software design, user-centered design

## Abstract

The lack of user-centered design principles in the current landscape of commonly-used bioinformatics software tools poses challenges for novice genomics researchers (NGRs) entering the modern genomics ecosystem. Comparing the usability of one analysis software to that of another is a non-trivial task and requires evaluation criteria that incorporates perspectives from both existing literature and a diverse, underrepresented user base of NGRs. To better characterize these barriers, we utilized a two-pronged approach consisting of a retrospective literature review of existing bioinformatics tools and prospective semi-structured interviews of the needs of NGRs. From both knowledge sources, the key findings that resulted in poor adoption and sustained use of most bioinformatics tools included: poor documentation, lack of readily-accessible informational content, challenges with installation and dependency coordination, and inconsistent error messages/progress indicators. Combining the results from the literature review and the insights gained by interviewing the NGRs, an evaluation rubric was created that can be utilized to grade existing and future bioinformatics tools. This rubric acts as a summary of key components needed for software tools to cater to the diverse needs of both NGRs and experienced users. Due to the rapidly evolving nature of genomics research, it becomes increasingly important to critically evaluate existing tools and develop new ones that will help build a strong foundation for future exploration.

## Introduction

Bioinformatics is a rapidly growing field that involves the application of statistical methods and computational algorithms to analyze and interpret biological data. With the increasing availability of genomics data, the development of bioinformatics tools has become essential for researchers to efficiently analyze and interpret the vast amounts of biological data generated. To rival this rapid growth of genomic data, the amount of bioinformatics tools being developed and published has increased dramatically (Clément et al., 2018). While most of those tools are developed in siloed laboratories, large scale analysis platforms have also risen in usage and popularity, such as National Human Genome Research Institute’s (NHGRI) Analysis Visualization and Informatics Lab-space (AnVIL)/Terra and the All of Us platforms (Terra, 2026; All of Us Research Program, 2026). Most of these tools, however, suffer widespread adoption challenges due to inherent usability barriers from a lack of user-centered design during developed and testing phases.

The International Organization for Standardization defines usability as “the extent to which a product can be used by specified users to achieve specific goals with effectiveness, efficiency and satisfaction in a specified context of use”. While fields like global health and healthcare education often hire professional design firms (such as IDEO or Dalberg) to tackle usability (Bazzano et al., 2017; Göttgens and Oertelt-Prigione, 2021), usability analysis in bioinformatics tools is scarce, posing barriers to proper development and sustained adoption (Bolchini et al., 2009; Carpenter et al., 2012). Tools created without usability considerations suffer from lack of sustained use (Pavelin et al., 2012; Mangul et al., 2019). Furthermore, academic software distribution challenges—such as assuming end-user technical backgrounds, relying on academic journals for distribution, and disseminating incomplete tools—pose great barriers for long-term stability.

To address these challenges, several bioinformatics tools have been developed with end-user needs in mind. Examples include GenomIUm, where expert bioinformaticians reported reduced complexity and easier analysis workflows, and the All of Us Cohort Selection Pipeline for streamlined case-control cohort selection (All of Us Research Program, 2026; Iñiguez-Jarrín et al., 2021). Larger research institutes like NHGRI and EMBL-EBI have also begun pushing for deliberate, user-friendly tool development, citing “the user is the future” (Pavelin et al., 2012; NHGRI Strategic Vision, 2020).

Although there is a push towards user-centered bioinformatics tools, novice genomics researchers (NGRs) are overlooked despite being keystone users. NGRs are individuals interested in conducting genetic experiments but lack quantitative backgrounds, bioinformatics experience, or access to extensive technological resources like local high-performance computational clusters. Examples of NGRs include new genomics trainees at academic institutions, researchers with limited computational experience, and scientists unfamiliar with modern genomics tools. As NHGRI’s core mission involves training a diverse genomic workforce, NGRs are a key user base for future tool adoption and sustained usage (NHGRI Strategic Vision, 2020; Funding for Research Training, 2026; Attwood et al., 2017). Machado et al. reinforces the information-gathering needs of novices and the hurdles faced: poorly designed databases, lacking documentation, and vague error handling (Machado et al., 2017). UCD principles can address these NGR needs, producing a workforce capable of effectively manipulating, analyzing, and sharing biological data (Al-Ageel et al., 2015).

Albeit limited, some work has begun to address NGR needs. Murphy et al. (2003) studied users who regularly navigated complex databases and designed a visual interface to ease the burden for novice users trying to find research patient cohorts (Murphy et al., 2003). Britto et al. (2009) conducted scenario based usability analysis of a pediatric patient portal to study the challenges faced by novice users and reported major barriers of medical language complexity and difficulties in navigation (Britto et al., 2009). We even see barriers of information overload and lack of proper tool documentation faced by non-specialists who try utilizing popular ontology creation tools to capture a relatively simple biological system (Recchia et al., 2020; Zhang et al., 2021). The challenges faced by novice users in the above studies are not limited to just the contexts of those investigations but are universal across bioinformatics as novice researchers continue to be overlooked.

To fill the gap of usability in bioinformatics tools and to query the needs of NGRs, we employed a mixed-methods study design consisting of two primary parts: a literature review and a needs assessment of target users. First, a retrospective analysis of existing bioinformatics analysis tools utilized by researchers to perform genetics experiments was conducted to better understand the current landscape of available analysis instruments. Thereafter, a prospective analysis using semi-structured interviews was conducted to assess the needs of novice genomics researchers from different sites. We hypothesized the existing literature and the initial needs of NGRs will primarily revolve around thorough documentation and tutorials, minimalistic user-interfaces, and low barriers of installation and deployment of bioinformatics tools.

## Methods

The literature review was conducted using guidelines set forth by Harris et al. (Harris et al., 2014). Given the scarcity of user-centered design (UCD) considerations in bioinformatics tool development (Pavelin et al., 2012), the review scope was intentionally broad to capture maximum literary evidence for the final evaluation rubric. To survey the existing landscape, the primary research question was: “What are the available bioinformatics analysis tools commonly utilized to conduct genetic analyses?” Table 1 lists the search strings utilized to identify relevant literature.

**TABLE 1:** Search criteria utilized to find relevant literature from different databases. Except for Pubmed, all other databases were manually filtered for the publication date (post 2003) and English language requirements.

Database	Search string
Pubmed	((“bioinformatic”) OR (“biomedical informatic”) OR (“genomic”) OR (“genetic”) OR (“informatic”) OR (“gene”)) AND ((“software”) OR (“tool”) OR (“platform”) OR (“analysis”) OR (“user-friendly”) OR (“UCD”)) AND (“2003” [Date - publication]: “3000” [Date - publication]) AND (English [Language])
Web of science	((“bioinformatic”) OR (“biomedical informatic”) OR (“genomic”) OR (“genetic”) OR (“informatic”) OR (“gene”)) AND ((“software”) OR (“tool”) OR (“platform”) OR (“analysis”) OR (“user-friendly”) OR (“UCD”))
Medline	((“bioinformatic”) OR (“biomedical informatic”) OR (“genomic”) OR (“genetic”) OR (“informatic”) OR (“gene”)) AND ((“software”) OR (“tool”) OR (“platform”) OR (“analysis”) OR (“user-friendly”) OR (“UCD”))
Bioinformatics software DB	Individual searches: “Genomic”, “gene”, “user-friendly”, “analysis”, “GWAS”
Google scholar	((“bioinformatic”) OR (“biomedical informatic”) OR (“genomic”) OR (“genetic”) OR (“informatic”) OR (“gene”)) AND ((“software”) OR (“tool”) OR (“platform”) OR (“analysis”) OR (“user-friendly”) OR (“UCD”))

After executing each search, abstracts were screened and excluded if they lacked informatics tools or genomic data, were conference abstracts, dissertations, not peer-reviewed, non-English, published pre-2004, or missing example results. Full articles were then excluded if they showed minimal software discussion, lacked tool URLs, omitted documentation details, or failed to identify potential users.

12 participants were recruited after receiving an exempt approval from the University of Washington Institutional Review Board (IRB) on 7 September 2022. This sample size aligns with estimates for thematic saturation in qualitative health research (Vasileiou et al., 2018; Braun and Clarke, 2013). Inclusion criteria were:

- Graduate student currently at the University of Washington or Tulane University.
- Prior experience with bioinformatics analysis methodologies.
- Prior experience with informatics tools and software.
- Less than 3 years of experience with genomic analyses.

The primary investigator (HP) created a semi-structured interview guide, which was refined after review by a UW qualitative expert and four total pilot interviews. DeJonckheere et al. have shown that semi-structured interviews offer an effective balance of rigor and flexibility for extracting qualitative themes (DeJonckheere and Vaughn, 2019). Scheduled for 45 min, each interview session included verbal consent, the interview, and a post-interview demographic questionnaire (also approved by the UW IRB). Audio recordings of the interview were stored on a secure UW Zoom-cloud and transcribed via HappyScribe (Scribe, 2026). After transcription, the output files were downloaded to a local ATLAS. ti project for analysis, and per security guidelines, all cloud-stored audio recordings and transcripts were permanently deleted (ATLAS.ti, 2026).

To analyze the transcripts for emergent qualitative themes, template analysis was conducted. Introduced by Crabtree et al., template analysis is a form of thematic analysis that utilizes hierarchical coding derived from a pre-defined template in addition to allowing for new codes that may appear from the transcripts (Crabtree et al., 1995). In the context of this study, the interview guide itself was utilized as the foundational basis for codebook development. The finalized codebook contained twelve hierarchical codes. These codes captured various aspects of the novice genomics researchers’ experience, including their first experiences getting into research, the resources used when getting into field, and the specific software tool(s) they utilized. Qualitative themes were categorized by participant reactions (emotional reaction versus cognitive reaction), specific tool attributes (benefits and barriers of specific tools), and broader consequences (impact on upkeep/cost, overall impact on research, and overall user experience). Finally, the codebook also included categories for participant feedback, namely, suggested improvements to bioinformatics tools and challenges/concerns with cloud genetics.

Before arriving at the final codebook and in order to maintain high inter-coder agreement, the primary investigator and an independent coder (JC) achieved a high Krippendorff’s Cu/cu-alpha coefficient of 0.919 (Krippendorff, 2011; Krippendorff, 2004) on four initial transcripts before independently coding the remaining transcripts. The raw and coded transcripts, along with the entire codebook and interview guide, can be found at https://github.com/hvpatel96/NGI_Needs_Assessment/.

To synthesize the knowledge gained from the literature review and needs assessment, an evaluation rubric was developed. Evaluation, within the context of professional assessment, is defined as the systematic determination of the quality or value of something (Scriven, 1991). A good evaluation requires knowledge about the evaluand (what you are evaluating), its background, and its context (Davidson, 2005). For this step, the evaluand was the effectiveness and usability of existing bioinformatics tools within the context of NGRs (Rubrics, 2026). Utilizing the methodology for evaluating bioinformatics tools and training practices from Xie et al. and Tracentenberg et al., an initial set of evaluation criteria were created to begin rubric creation (Xie et al., 2021; Tractenberg et al., 2019). These criteria were then adjusted to incorporate the needs of NGRs and the strengths and weaknesses of bioinformatics tools gathered from the literature review. The primary investigator then utilized Markiewicz et al.‘s primer on evaluation framework development to create a scoring metric for each criteria (Markiewicz and Patrick, 2016).

## Results

After querying the literature databases, we identified 385 potential studies. Upon removing duplicates (196) and screening titles/abstracts (133), 56 full-text studies remained. Of these, 22 were excluded due to lacking genomic tool availability, documentation, or target audience information. Ultimately, 34 studies remained for the literature review.

Of the genomics analysis tools from the 34 studies, 32 (94%) had documentation/tutorial content provided, 17 (50%) had a graphical user interface, 8 (24%) were cloud-compatible, 8 mentioned some UCD considerations (24%), and only 7 (21%) studies mentioned a target audience of novice users.

A total of 12 participants were interviewed, with a mean age of 27 years (SD 7, range 21–48). The cohort was predominantly female (58%, N = 7) and non-Hispanic/Latino (92%, N = 11). The racial breakdown was 50% Asian (N = 6), 33% White (N = 4), and 8.5% each for multi-race and other/non-specified. Highest education level was split between Bachelor’s (50%) and Master’s (42%) degrees, with one participant holding a Doctor of Medicine degree (8%). Reflecting their status as novice researchers, 75% had less than 2 years of experience working with genetic data (33% less than 1 year, 42% 1–2 years), and the median self-reported familiarity with genomics was 3 out of 5.

Participants’ emotional reactions varied when first utilizing bioinformatics tools. Some reported satisfaction and excitement:

“Well, I was super excited about it, actually. This was actually my first time that I got to look at real data and trying to figure out how to use it and make it useful.” (P12)

However, the primary emotions shared by the majority of the participants were frustration, confusion, and being overwhelmed:

“Honestly, that was terrible for me … a starter, the documentation and the reason you run through the pipeline [PLINK] is it's really hard, like, for a starter to figure out every component in that. So that's going to cause a lot of headache.” (P2)

These emotions mirrored cognitive reactions regarding initial concerns. Most lacked the domain and technical knowledge required to utilize their tools:

“So, I think that it [Bioconductor GWAS] feels a lot of them feel like a black box, essentially, where at least in my experience, you do not know what's going on. You do not know if it's working. You put the arguments in and you pray, and then when it finishes, you'll see if it worked or not.” (P6)

“It definitely caused a lot of frustrations because of their documentation and some of those error messages. It's not really clear. When you also Google that [the errors] there are a bunch of other people googling the same things you could find and the answers are actually various.” (P2)

Conversely, a few participants reported sufficient initial comprehension, which improved after following tutorials or prolonged use:

“It [AllOfUs] did not really have anything that I thought that they were missing. I thought they were pretty clear on how to use everything and they're very informative. And then they also add a more detailed help support system in terms of like, if you did get lost in trying to understand how to use one of their specific tasks that they had.” (P5)

Positively, participants appreciated provided tutorials, documentation, tool robustness (even with large datasets), and built-in privacy and security security precautions:

“I think the biggest plus for me is that it has very robust documentation. It still has some holes in it, I think, but the least of the software tools that I was using. At least it's pretty simple to go from zero to being able to use it.” (P6)

Conversely, negatives included trouble during initial setup, lack of thorough error messages, lack of user-friendly interfaces, and challenges sharing results outputted to colleagues or other pipelines:

“I think often times some tools are set up in a way where it's not immediately evident what you need to do. It was frustrating because you would submit something and you would not know for a while whether or not it worked. And so then you get the error or whatever a few hours later and then you have to debug and you feel like you've lost a few hours.” (P8)

Despite entry barriers, most participants benefited from learning these tools. Recommendations for improvement (9/12 participants) heavily focused on clearer error messages, interactive environments, and better documentation written in layman’s terms:

“I expected more documentation on the whole thing. They have some documentation, but they do not have many examples. So I think they could really improve in that regard.” (P3)

“I would definitely wanted them to provide more examples of the workflow and how the tutorial would go. Like, if you were doing some genomic analysis and you need to say grab or filter specific regions, what do you do and what do you most of the frequently asked questions or errors.” (P2)

Four participants requested an integrated “information hub” during analysis:

“Honestly, I wish any tool's methods paper had, like, explain it like I'm five section or something. Because, man, some methods papers are just so convoluted. Also, I think I would have wanted the tool to like point to useful resources, like useful background reading.” (P8)

Regarding the shift toward cloud computing, participants were generally optimistic but raised concerns regarding privacy, security, cost, and required training:

“I think there are a couple of things I mentioned already. What is the cost structure of it? What do I need to know to set it up correctly from a technical perspective? Like if you're using health data, you have to think about HIPAA and proper access control.” (P12)

Table 2 presents the evaluation rubric, adapted from existing bioinformatics criteria and refined using insights from the literature review and needs assessment. It covers analysis robustness, documentation, data restrictions, accessibility, reproducibility, tool interoperability, and user-centered design.

**TABLE 2:** Evaluation Rubric for Bioinformatics Tools within the context of NGRs.

Evaluation rubric for bioinformatics tools	Level 0	Level 1	Level 2	Level 3
Criteria	Unacceptable	Marginal	Acceptable	Exceptional
Robustness of genetic analyses	Only capable of one very specific type of genetic analysis (fixed parameters) and lacks ability to scale	Capable of one or two specific analyses but able to scale to large datasets	Supports many genetic analyses, can scale to large datasets, but lacks clear vision for use of tool	Many genetic analyses supported or able to be supported (users can create/share workflows), clear vision for tool’s usage
Tutorials/Documentation	Had little/no documentation or tutorials about how to setup or utilize tool to perform analyses	Had documentation/tutorials but was difficult to understand/outdated	Had documentation and tutorials but lacked sample data or references to publications	Clear, updated documentation with examples and sample data along with links to publications that utilized the analysis tool
Data restrictions	Supported only one or two specifically formatted data types	Supported specific formatted data types but did not allow use of new data	Supported specific formatted data types and allowed the upload of new data	Supported robust data types and allowed for the upload of new data
Accessibility (cloud/Free-to-use/Open-source)	Inaccessible to the target userbase, behind paywall, proprietary code with little option of how to use (their own platform)	Somewhat accessible to the target audience, not open-sourced, may not be free-to-use	Mostly accessible to the target userbase (some wait if user needed to be validated), some open-source components, and free-to-use	Very accessible tool with both local and cloud-based options, open-source and free-to-use
Reproducibility/Interaction with other tools	Does not have built in functionality for reproducing experiments or interacting with other software tools	Has built functionality for reproducing experiments but does not interact with other software tools	Has reproducibility functionality built-in and able to interact with other tools but in a very limited range	Excellent built in features for reproducing experiments and is able to interact with other tools with clear guidelines and input/output types
UCD design considerations (setup, usage, evaluation)	Had little to no usability considerations during the design, development, or evaluation of genetic tool	Had some usability considerations but only during one or two phases of the developmental cycle	Had some usability considerations during most of the development phase but lacked clear interaction with the target user base	Utilized usability considerations during all stages of the development process with clear interaction with target userbase
Overall performance	Unacceptable	Marginal	Acceptable	Exceptional
Points required	0–6	6–11	12–16	16–18

## Discussion

This investigation broadly captures existing challenges in bioinformatics software tools, including missing documentation, setup difficulties, reproducibility issues, and a lack of user-centered design (UCD) considerations. Using focused literature review and user interviews, we collected curated data that offers nuanced perspectives on why these challenges exist, how challenges relate to one another, and potential solutions for future tools.

The literature review identified common characteristics among widely used and highly cited genomic software over the past 2 decades. While most tools were open-source with some tutorial content, very few noted a target audience or incorporated UCD components. Tools mentioning UCD principles did so briefly, without active incorporation during design and deployment. Studies have shown that poor usability considerations in shared instruments often lead to a failure of sustained use (Bolchini et al., 2009). Furthermore, as only 7 of 34 evaluated tools mentioned novice users, most widely used genomics tools fail to align with the NHGRI’s 2020 Strategic Vision for breaking down barriers and building a robust research foundation (NHGRI Strategic Vision, 2020).

User interviews detail the challenges novice genomics researchers (NGRs) routinely face during software setup, exploration, experimentation, and sustained use. Almost all interviewed NGRs reported feeling overwhelmed during initial adoption, struggling to find adequate learning resources due to lacking domain expertise or technical knowledge. Participant 6 sums it up nicely:

“So, I think that it [Bioconductor GWAS] feels a lot like a black box, essentially, where at least in my experience, you do not know what's going on. You do not know if it's working. You put the arguments in and you pray, and then when it finishes, you'll see if it worked or not.” (P6)

This sentiment is supported by participants’ calls for detailed documentation, accessible tutorials, and clearer error messages. While solutions for novice users exist in other disciplines, bioinformatics software introduced to NGRs lacks the polish needed to support them (Pavelin et al., 2012; Attwood et al., 2017).

Additionally, most participants reported challenges in result reproducibility and shareability. This crisis is especially critical for NGRs, causing increased frustration, delayed software adoption, and difficulty connecting theoretical concepts to practical applications. Potential future solutions include integrating workflow description language (WDL), saving rerun-ready prior experiments, providing guided documentation, and offering external support (e.g., emails or forums). Furthermore, integrating artificial intelligence (AI) assistants to offer guided code generation and error resolution could alleviate many of the usability barriers noted above, though these technologies also raise concerns regarding data privacy and code transparency.

Combining literature review findings and NGR interviews, an evaluation rubric was created to grade existing and future bioinformatics tools. This rubric summarizes key components required to cater to diverse user needs. As detailed in Table 2, user interviews indicate that UCD considerations during setup and detailed supplementary information are paramount for designing usable software. Existing studies efficiently utilizing these components report higher throughput, better user understanding, and sustained tool use (Murphy et al., 2003; Recchia et al., 2020; Zhang et al., 2021). Therefore, critically evaluating existing tools and developing new ones is increasingly important for building a strong foundation for future genomics exploration.

This study has several limitations. The literature review only included English articles, excluding software published in non-English journals. Incorporating additional peer-reviewed sources or reinterpreting the research question could yield different final studies. Although user interviews yielded rich qualitative data, the participant pool was relatively small. However, theoretical saturation was reached, as code groups and themes did not change significantly after 10 participants. Participants were also limited to the University of Washington and Tulane University and were mostly of self-identified White and Asian descent; their training may not reflect the views of NGRs elsewhere. Future investigations should include a more diverse participant pool. Finally, recognizing the subjective nature of qualitative research, the primary investigator’s (H.P.) analysis represents only one possible interpretation of the data.

This study underscores a pivotal opportunity for the field of bioinformatics: by embracing user-centered design and addressing the needs of novice genomics researchers, we can build tools that are not only more accessible but also more impactful. Improving usability, documentation, and reproducibility will empower a broader, more diverse community of researchers to contribute to genomic discovery. As genomics continues to evolve, prioritizing usability ensures that innovation is not limited by expertise, but expanded by it, fueling a more collaborative and transformative future.

## Data Availability

The raw data supporting the conclusions of this article will be made available by the authors, without undue reservation.
